# A Public Health Framework to Improve Population Health Through Health Care and Community Clinical Linkages: The ASTHO/CDC Heart Disease and Stroke Prevention Learning Collaborative

**DOI:** 10.5888/pcd16.190065

**Published:** 2019-09-12

**Authors:** Rose Anne Felipe, Marcus Plescia, Emily Peterman, Holly Tomlin, Michael Sells, Camillia Easley, Kaha Ahmed, Letitia Presley-Cantrell

**Affiliations:** 1 Association of State and Territorial Health Officials, Arlington, Virginia; 2 Tomlin Health Sciences Communications, Brooklyn, New York; 3 Centers for Disease Control and Prevention, Atlanta, Georgia

## Abstract

Thirty-one state and territorial public health agencies participated in a learning collaborative to improve diagnosis and management of hypertension in clinical and community settings. These health agencies implemented public health and clinical interventions in medical settings and health organizations using a logic model and rapid quality improvement process focused on a framework of 4 systems-change levers: 1) data-driven action, 2) clinical practice standardization, 3) clinical–community linkages, and 4) financing and policy. We provide examples of how public health agencies applied the systems-change framework in all 4 areas to assess and modify population-based interventions to improve control of hypertension. This learning collaborative approach illustrates the importance of public health in the prevention and control of chronic disease by supporting interventions that address community and clinical linkages to address medical risk factors associated with cardiovascular disease.

**Summary**

**What is already known on this topic?**

Integrating public health, clinical care, and community approaches can improve the clinical, social, and economic burdens of cardiovascular disease.

**What is added by this report?**

A learning collaborative to support state and territorial health agencies, health care systems, and community partners was developed to implement evidence-based practices for hypertension diagnosis and control across communities. A systems-change framework and rapid quality improvement process helped increase coordination between health agencies and health care systems.

**What are the implications for public health practice?**

This learning collaborative shows that health agencies in various jurisdictions can improve communication between community health organizations and public health and leverage technical and financial resources to support programs for patients to self-manage their blood pressure.

## Background

Cardiovascular disease (CVD) is the most common cause of death in the United States and a source of suffering and disability. Reductions in deaths from CVD are largely due to decreased use of tobacco products, improvements in blood pressure and cholesterol control, and advances in medical treatment (1,2). However, declines in death rates from heart disease have slowed, and additional action is needed to sustain progress and decrease the risk of illness and death associated with CVD (3).

In 2017, the American College of Cardiology and the American Heart Association released new blood pressure guidelines that suggest lowering the optimal blood pressure target from below 140/90 mm Hg to below 130/80 mm Hg; however, among people with high blood pressure in the United States, only half were in compliance with the previous guidelines, and fewer will meet the new ones (4). Diagnosis and control of hypertension is an opportunity for public health entities to work with health care systems at the state, tribal, local, and territorial levels to support and improve clinical care of patients with high blood pressure. In addition, blood pressure control largely depends on patient self-management and may benefit from more comprehensive community-based approaches (5–7).

## Cooperative Agreement Purpose and Structure

In 2013, the Centers for Disease Control and Prevention (CDC), Division for Heart Disease and Stroke Prevention, began a cooperative agreement with the Association of State and Territorial Health Officials (ASTHO), a national nonprofit organization representing public health agencies in the United States, the US territories, and the District of Columbia. ASTHO and CDC developed a learning collaborative, defined as a group of public health leaders and partners who have a common interest in a subject area that collaborates to achieve sustainable change and improvement. The ASTHO/CDC Heart Disease and Stroke Prevention Learning Collaborative was designed to support state and territorial health agencies, health care systems, and community partners in efforts to improve hypertension diagnosis and control in and across communities by supporting the implementation of evidence-based practices (8).

ASTHO and CDC developed a logic model for the learning collaborative that served as a blueprint for health improvement measures and approaches (Figure). The logic model was based on the CDC National Center for Chronic Disease Prevention and Health Promotion’s 4 domains of public health action (9). A framework for systems change was developed for the learning collaborative (10), and it focuses on 4 systems-change levers: 1) data-driven action, 2) clinical practice standardization, 3) clinical–community linkages, and 4) financing and policy (10).

**Figure:**
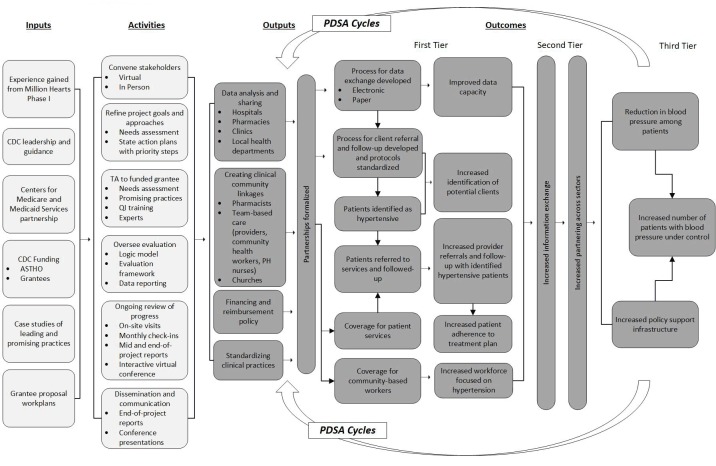
Logic model for ASTHO/CDC Heart Disease and Stroke Prevention Learning Collaborative. Abbreviations: ASTHO, Association of State and Territorial Health Officials; CDC, Centers for Disease Control and Prevention; PDSA, Plan, Do, Study, Act; PH, public health; QI, quality improvement; TA, technical assistance.

Jurisdictions, including state, territorial, and tribal-serving organizations, partnered with local public health agencies, community health centers, and private clinics to implement programs that prevent, detect, and reduce hypertension rates. States, tribal-serving organizations, and territories developed an aim statement to summarize their project’s intervention approach, objectives, and program, which provided a source of continuity of reporting and data sharing, as officials and partners regularly communicated and reported on project progress to CDC, ASTHO, and other jurisdictions in their cohort. Jurisdictions used the logic model as a guide to help categorize their intervention approaches into the 4 main components of the ASTHO framework. The learning collaborative also used a rapid quality improvement process focused on the “plan,” “do,” “study,” and “act” (PDSA; Institute of Health Improvement [11]) model to improve program implementation in a rapid, yet systematic fashion. The PDSA cycle allowed for rapid implementation, refinement of activities in the framework, and process improvement in a 10-month period.

We reviewed our experience using a 4-component framework to address systems change and the rapid quality improvement process to help states change systems, provide an overview of activities from 3 state health departments who implemented the framework, and summarize the implications for public health practice of using this approach.

## Implementation of the Learning Collaborative

After an ASTHO-led Request for Proposal, states, tribal-serving organizations, and territories (jurisdictions) submitted proposals and were funded to develop a quality improvement process to improve hypertension diagnosis and control (Phase 1) (9). Ultimately, 31 jurisdictions, which included partnerships with tribal-serving organizations, participated in the learning collaborative during a 5-year period. Each collaborated with a range of stakeholders, which included public health agencies, health care providers, clinical quality improvement organizations, health information technology experts, public and private payers, pharmacists, community-based health care professionals, community organizations, local health departments (LHDs), and others. These unique partnerships provided jurisdictions with access to various resources to facilitate community and patient engagement (eg, home blood pressure monitoring and pharmacy counseling programs), as well as data sources (eg, electronic patient registries) to identify people with undiagnosed hypertension, uncontrolled hypertension, or both.

We applied the strategies used to implement the ASTHO/CDC framework in 3 states, each with a unique set of characteristics: New York, Oklahoma, and Arkansas (Table). New York State has many resources and has consistently been an early adopter in implementing population-based interventions through its health department to improve control of medical risk factors for chronic disease. New York participated in Phases 1 and 2 of the learning collaborative. Arkansas has modest resources and has emerged as a leader in addressing community–clinical linkages to address self-management of chronic disease risk factors through work with local clinics. Arkansas enrolled during the second year (Phase 2) and continued to participate throughout the 5 years. Oklahoma instituted a unique collaboration between the state health department and an independent, self-governed, tribal nation. Oklahoma joined the collaborative during the third year (Phase 2) of implementation under an expansion of the initiative to address hypertension disparities in American Indian/Alaska Native populations. Each state used a comprehensive approach to improve hypertension identification and control by working across all 4 systems-change levers in our framework (Table). Each state excelled in its implementation of specific areas of the framework.

**Table:** State and Tribal Characteristics and Results of Evidenced-Based and Promising Best Practices in 3 States, ASTHO/CDC Heart Disease and Stroke Prevention Learning Collaborative, 2013–2018

Best Practices Used to Achieve Results	New York State	Oklahoma	Arkansas
**Community–clinical linkages**			
Establish connections between health care, public health, and other jurisdictions to improve access to hypertension services and support throughout the care continuum, as well as increase data sharing among states and territories.	Local health departments and Federally Qualified Health Centers; home blood pressure monitoring program with clinical support; health information exchange data analysis.	Pharmacy hypertension clinic; Choctaw Nation health system and pharmacists; academic partnership with University of Oklahoma Health Sciences Center College of Pharmacy.	Partnerships with providers, local health units, community pharmacies and senior centers in rural, underserved communities.
**Data-driven action**			
Improve data exchange or capacity by using health information technology to facilitate patient identification, referral, and follow-up.	Metrics developed with electronic medical record data; data registries used to track and contact patients; data system used to evaluate and report clinical outcomes.	Data from electronic health records used to identify patients with uncontrolled hypertension for referral (counseling or management).	Used data from electronic medical records to identify undiagnosed hypertension. Partnership with Humana to improve quality of care.
**Standardization of clinical practice**			
Implement protocols to ensure consistency in intervention implementation and data collection and analysis methods.	Adopted and implemented hypertension treatment protocols; home blood pressure program with clinical support; and systems for tracking and follow-up.	Developed a referral process; established a pharmacist–provider collaboration; educated and counseled patients; calculated arteriosclerotic cardiovascular disease risk; and conducted blood pressure monitoring and follow-up.	Protocols for referrals to local clinics established a program for counseling by pharmacist; developed strategies for hypertension management based on a team-based care framework.
**Financing and policy**			
Create a sustainable system to improve hypertension prevention, detection, and control through payment reform, and help jurisdictions leverage funding outside of the learning collaborative to establish systems of care or expand their programs and initiatives to other areas throughout the jurisdiction.	Instituted a 90-day pharmacy benefit to expand coverage for medications for hypertension in their Medicaid-managed care plans.	Computed a return of investment of $160 per dollar spent, based on the average emergency department cost of a single cardiovascular disease event.	Established a partnership with a private payer, a health care coalition, and a hospital to develop a payer model for transition of hypertension care from emergency departments to team-based care and medical homes.

Abbreviations: ASTHO, Association of State and Territorial Health Officials; CDC, Centers for Disease Control and Prevention.

**New York.** New York used data-driven action to support Federally Qualified Heath Center use of electronic health records to identify and track patients with hypertension, resulting in an improvement in hypertension control rates of 18.7% across centers in just 2 years. Their use of a regional health information exchange provided real-time county-level rates of hypertension, hypertension control, and undiagnosed hypertension and is now a model for other state and territorial programs.

**Arkansas.** Arkansas developed and tested well-defined hypertension care management plans in 4 counties, on the basis of a community team-based care approach that ultimately became the model for a standardized protocol that is now used statewide. They used a web-based pharmacy platform to help community pharmacists identify patients with uncontrolled blood pressure and calculate and monitor patient medication adherence (12).

**Oklahoma.** A unique collaboration with Oklahoma and the Choctaw Nation leveraged community resources to establish a pharmacy-based hypertension management model through a partnership with a university college of pharmacy. The approach greatly expanded self-management options and resources for patients across a large rural area, throughout nontribal health systems and within the Choctaw Nation.

## Implications for Public Health Practice

The jurisdictions participating in the ASTHO/CDC Heart Disease and Stroke Prevention Learning Collaborative are compelling examples of effective approaches to hypertension management and control that can be implemented at the state and community levels when funding and technical support are made available. Before their participation in the learning collaborative, states received direct funding from CDC to support their core heart disease and stroke prevention programs. The learning collaborative work built on this capacity and provided a structured environment for states to work more deliberately on systems change using the team-based rapid improvement model. Learning collaborative states received modest additional funding through the learning collaborative, which was used to facilitate team building, expand data collection efforts, and support additional reporting requirements. Grantees were able to hire additional personnel to oversee and facilitate their intervention approach, expand the use of jurisdiction-wide standardized hypertension measures, refine and expand capacity to use health information exchanges to inform clinic-based and population-based health improvement efforts, and establish and strengthen ongoing, standardized clinical data reporting.

Findings from these case studies support early reports that integration of public health, clinical care, and community health centers can help health systems address the clinical, social, and economic burdens of CVD (8,9). These jurisdictions demonstrated short-term gains in health systems integration and progress toward long-term goals of systems and policy change to improve hypertension diagnosis and control.

This learning collaborative illustrates how public health efforts are necessary to help prevent and control chronic disease by supporting interventions that focus on clinical outcomes associated with CVD. Although clinical outcomes are challenging to attribute to a population health program, in part because of a lack of a comparison group, quality improvement programs have shown substantial improvements in management and control of chronic diseases when public health and clinical care services are integrated (10,13). However, implementing large program-based initiatives typically takes time, is contingent on both public and private partnerships, and requires multiple resources for implementation and evaluation. ASTHO was able to accelerate the implementation process, while maintaining standards for quality improvement because of its ties with jurisdiction health leaders and historical success with multisector collaboratives (8,9). A focus on a 4-component framework of systems-change levers, and a rapid quality improvement process allowed for increased coordinated efforts between jurisdictions and community health agencies. Jurisdictions had opportunities to assess the progress of their intervention, rapidly adjust their program with tools provided by ASTHO and CDC, and share evidence-based best practices among other jurisdictions.

Blood pressure control is largely dependent on patient self-management programs. However, such programs are less frequently integrated into the team-based care model and monitored by health care systems (5–7). This learning collaborative shows that health agencies in various jurisdictions can facilitate communication between community health organizations and public health and leverage technical and financial resources to support programs for patients to self-manage their blood pressure. Ultimately, other funding streams and strategies such as health care payer reimbursement are needed to sustain these programs and take them to a national scale.

Jurisdictions that participated in the ASTHO/CDC Heart Disease and Stroke Prevention Learning Collaborative addressed different intervention approaches. These approaches included partnering with leadership from traditionally marginalized communities, bridging clinical services, and providing capacity building. Our report highlights a framework of systems-change levers that addresses key areas for program sustainability and reach. Examples include using electronic health record systems to drive identification of undiagnosed and uncontrolled hypertension; implementation of protocols for treatment, referrals, and follow-up to ensure clinical practices are standardized across public health; and formation of partnerships between community organizations and local clinics that help expand networks and self-management support. An evaluation of the approaches and outcomes of the 5-year learning collaborative is under way (unpublished data). It will provide further insight into differences in governmental public health structures that may better integrate diagnosis and control of hypertension at the community level and improve outcomes.
